# Research progress of copper in diabetes and diabetic kidney disease: a narrative review

**DOI:** 10.3389/fendo.2025.1584084

**Published:** 2025-10-16

**Authors:** Zongheng Wu, Sumei Li

**Affiliations:** ^1^ The Graduate School of Fujian Medical University, Fuzhou, Fujian, China; ^2^ Department of Endocrinology, The First Hospital of Putian City, Putian, Fujian, China

**Keywords:** copper, blood copper, urinary copper, diabetes, diabetic kidney disease

## Abstract

Copper is a trace element necessary for the normal growth and development of the human body and is involved in a wide range of physiological processes. However, copper has a dual role in the body: copper deficiency impairs the function of copper-binding antioxidant enzymes, while copper excess can lead to cell damage and even cell death by promoting oxidative stress. Studies have shown that there is a link between copper levels and diabetes, which has been implicated in glucose metabolism and the progression of diabetic complications through a variety of mechanisms. Diabetic kidney disease (DKD), a serious complication of diabetes mellitus (DM), lacks effective prevention and treatment. As a result, the relationship between copper and DKD is beginning to attract the attention of researchers. However, inconsistencies in the results of studies to date suggest that the mechanisms underlying the relationship between copper and diabetes may be more complex. This review summarizes the relevant research progress on the relationship between copper and DM and DKD, with the aim of providing new perspectives and references for research in related fields.

## 1 Introduction

Today, diabetes has become one of the major chronic diseases threatening human health. The global prevalence of diabetes is on the rise, with prevalence among people aged 20–79 years projected to increase from 10.5% (536.6 million people) in 2021 to 783.2 million people in 2045 (1). Diabetes mellitus (DM) is primarily characterized by chronic hyperglycemia resulting from impaired insulin production or function and is associated with a variety of serious complications (2). Diabetic complications are mainly divided into two categories: microvascular lesions, which include diabetic kidney disease (DKD), diabetic retinopathy, diabetic cardiomyopathy, and diabetic peripheral neuropathy; and macrovascular lesions, which include cardiovascular disease, cerebrovascular disease, and arterial lesions of the lower extremities (3). Among these complications, the epidemiologic characteristics of DKD show a significant increase in incidence and prevalence over the past decade, with approximately 20%-40% of the world’s 4.63 billion diabetic population suffering from DKD (4). DKD dominates the etiologic composition of end-stage renal disease (ESRD) in developed countries, with its share reaching 30-50%, making it the most important causative factor (5). Although existing treatments (e.g., glycemic control and reduction of urinary albumin) have slowed the progression of DKD to some extent, they have not yet been able to fundamentally alter its course (6). Therefore, it is important to explore new treatment strategies.

Copper (Cu) is an essential trace element that is important for human metabolism and homeostasis of the internal environment (7). It acts as a cofactor for a number of copper-containing enzymes (e.g., cytochrome c oxidase, Cu/Zn superoxide dismutase (Cu/Zn-SOD), and dopamine monooxygenase) and is a component of many important biological processes, one of which is the antioxidant defense system (8). It is also important to maintain optimal copper levels in the body, as excess or deficiency can be detrimental to cellular health (9). A growing body of studies have demonstrated a close association between copper levels and the development of diabetes and its associated complications, including DKD (10). However, the evidence for a link between copper levels, diabetes, and DKD remains controversial (11). This narrative review was searched on PubMed using the keywords “copper, blood copper, urinary copper, diabetes, diabetic kidney disease, copper chelators”. The authors searched the literature on “copper and diabetes” for the last three years. The literature on “Copper and DKD” and “Copper chelators and DKD” was searched for the last decade or more, as there are relatively few studies in this area. One author (Z.W.) selected relevant articles for review based on title and abstract, which was strictly supervised by the second author (S.L.). The purpose of this review is to provide a comprehensive summary of the latest research on the relationship between copper and diabetes. It also seeks to review the existing literature on copper and DKD, with the aim of providing a reference point and basis for future studies in this area.

## 2 Copper homeostasis

### 2.1 Copper absorption

The absorption of dietary copper, which exists primarily in the Cu²^+^ form, occurs mainly in the duodenum, with the stomach and distal ileum also contributing to a lesser extent (12). Prior to absorption by enterocytes, Cu²^+^ must be reduced to Cu^+^. This reduction is facilitated by key proteins such as the six-transmembrane epithelial antigen of the prostate (STEAP) and duodenal cytochrome B (DCYTB). Subsequently, Cu^+^ is taken up into the enterocytes through the high-affinity copper transporter 1 (CTR1, encoded by the SLC31A1 gene), located on the apical membrane (13).

### 2.2 Copper transport

Following its entry into the enterocytes, copper within the cytoplasm is delivered to specific organelles or target proteins via dedicated copper chaperone proteins. Alternatively, it can be sequestered for storage by binding to metallothionein (MT) (14). When the intracellular copper concentration rises, the ATPase copper-transporting alpha (ATP7A) is activated and relocates to the basolateral membrane of the cell, where it pumps copper out of the enterocyte (15). The exported Cu^+^ is rapidly bound by soluble chaperones in the blood—including albumin, transcuprein, histidine, and α2-macroglobulin—and transported to the liver via the portal venous system (16).

### 2.3 Copper distribution and excretion

**Figure 1:**
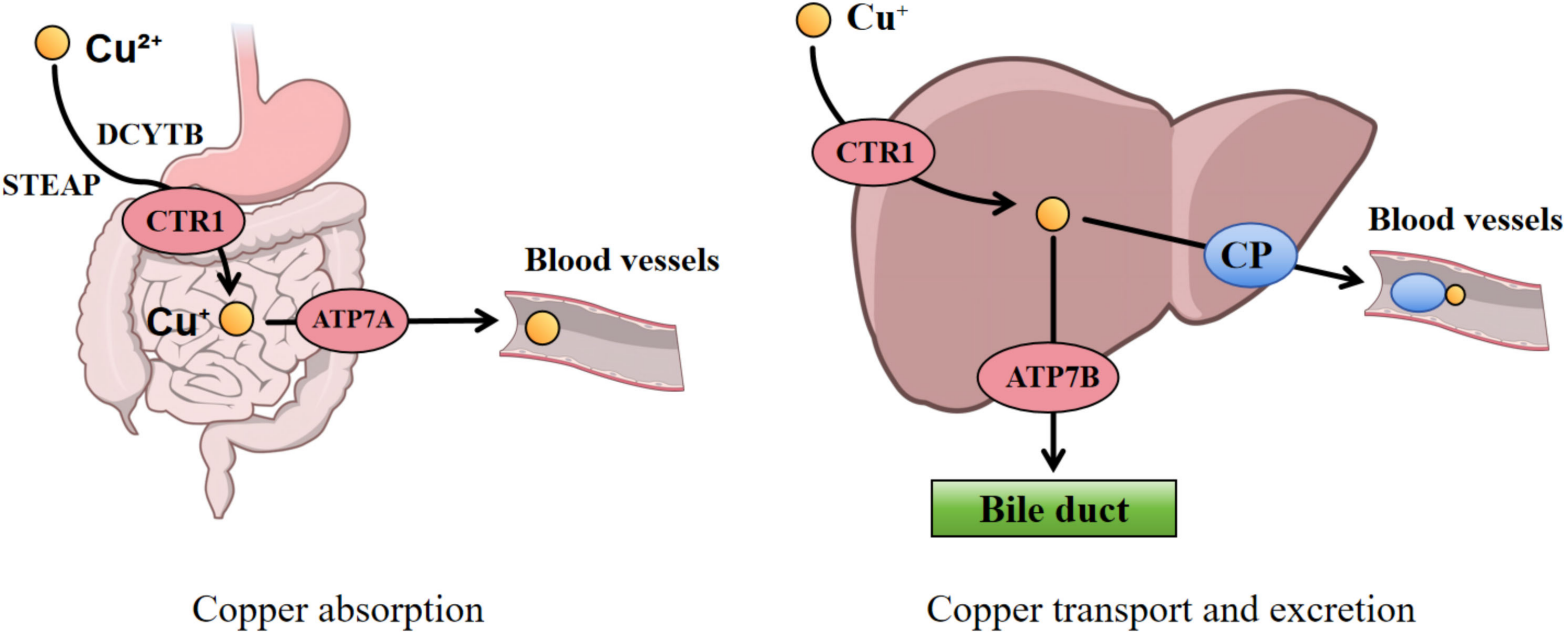
Systemic copper homeostasis. Dietary copper (Cu²^+^) is reduced to Cu^+^ by STEAP/DCYTB in the duodenum and subsequently absorbed via the copper transporter CTR1. Copper is exported into the portal vein via ATP7A, where it is bound to albumin for transport to the liver. Within hepatocytes, copper is incorporated into ceruloplasmin for systemic distribution or excreted into bile via ATP7B. STEAP, six-transmembrane epithelial antigen of the prostate; DCYTB, duodenal cytochrome B; CTR1, copper transporter 1; ATP7A, ATPase copper-transporting alpha; ATP7B, ATPase copper-transporting beta.

The liver is the central organ regulating systemic copper homeostasis. Hepatocytes uptake copper from the blood via CTR1 located on their cell membrane (17). Once inside the hepatocyte, copper follows two primary pathways: firstly, it is delivered to specific target proteins by copper chaperones (e.g., COX17, ATOX1, CCS) to participate in physiological processes such as mitochondrial energy production (cytochrome c oxidase) and antioxidant defense (superoxide dismutase 1, SOD1) (18). Secondly, copper is incorporated into ceruloplasmin, a crucial plasma copper transport protein. Copper bound to ceruloplasmin is secreted into the bloodstream and delivered to various tissues and organs (e.g., the brain), where it catalyzes numerous physiological reactions including neurotransmitter metabolism, redox balance, and extracellular matrix remodeling (19). Hepatocytes utilize another key transporter, ATPase copper-transporting beta (ATP7B), to pump excess copper into bile for ultimate elimination from the body, representing the primary excretory pathway for maintaining copper balance. Additionally, a small amount of copper is excreted through urine and sweat (15, 20) (**Figure 1**).

### 2.4 Disorders of copper metabolism and diabetes

Studies have shown that hyperglycemia can upregulate the copper transporter CTR1 in endothelial cells via the ASH2L-STEAP4 axis, enhancing Cu^+^ influx and contributing to diabetic endothelial dysfunction, thereby promoting the development of diabetic vascular complications (21).

In STZ-induced diabetic mouse models, reduced expression of ATP7A in vascular smooth muscle cells impairs copper efflux, leading to intracellular copper accumulation and subsequent vascular endothelial dysfunction. This phenomenon appears directly linked to insulin deficiency, as exogenous insulin intervention can restore ATP7A expression levels (22). Furthermore, myocardial copper deficiency has been observed in STZ-induced type 1 diabetic rats. Insulin insufficiency decreases ATP7B protein expression and disrupts its normal subcellular localization in cardiomyocytes, further compromising myocardial copper homeostasis (23).

These findings indicate that under diabetic conditions, both hyperglycemia and insulin deficiency can disrupt cellular copper metabolic balance by differentially regulating the functions of CTR1, ATP7A, and ATP7B, ultimately driving the progression of diabetic complications.

## 3 Copper and diabetes

### 3.1 Dietary copper intake and DM

Copper is an essential trace element and the main dietary sources are animal offal, nuts, fruits and cereals (24). The significant upward trend in the incidence of type 2 diabetes mellitus (T2DM) in recent years is thought to be closely related to changes in dietary patterns. Intervention strategies based on lifestyle modification, including changes in diet and physical activity, have been shown to be effective in reducing the risk of diabetes (25). Thus, the relationship between dietary copper intake and DM has gradually become a hot research topic.

Kim et al. investigated the relationship between dietary intake of micronutrients (including iron, copper, and zinc) and the risk of T2DM through a dietary survey of 16,666 participants and found that dietary intake of iron, copper, and zinc may be positively correlated with the risk of T2DM (26). From the perspective of type 1 diabetes mellitus (T1DM), Basu et al. examined the correlation between dietary copper intake and blood glucose levels. It was found that dietary intake of copper was positively correlated with glycated hemoglobin (HbA1c) levels. This suggests that dietary copper may play an significant role in glycemic control in T1DM and non-diabetic controls, and that excessive dietary copper intake may lead to poor glycemic control (27). Copper has been shown to participate in glucose metabolism through various mechanisms, including regulation of glucose transport and insulin synthesis and secretion (28). The above findings further support the potential importance of copper in the progression of diabetes.

Studies have shown that imbalances in copper and zinc ion homeostasis can lead to disturbances in the body’s oxidative-antioxidant system and adversely affect the function of pancreatic islet, thereby triggering or exacerbating the pathological process of diabetes and its complications (29), and its deficiency disrupts insulin homeostasis *in vivo*, resulting in decreased insulin secretion by beta cells (30). An imbalance between oxidants and antioxidants is a major cause of the development of diabetic complications, and copper and zinc are trace elements vital for the normal activity of Cu/Zn-SOD, an important antioxidant defense system (31). Since the *in vivo* balance of zinc and copper is closely related, such as competing for absorption in the gastrointestinal tract as well as sharing the same transport proteins (32), a simultaneous study of the effects of both on diabetes mellitus is needed. In a study by Laouali et al, the link between dietary copper/zinc ratios and the prevalence of T2DM was investigated from the perspective of dietary intake. It was found that there was a positive relationship between dietary copper/zinc ratios and the risk of T2DM, further demonstrating that the balance of copper and zinc has a considerable impact on the risk of diabetes (33).

### 3.2 Blood copper and diabetes

However, due to possible recall bias in dietary surveys and individual differences in copper intake, absorption and metabolism, A growing number of studies have begun to investigate the relationship between blood copper levels and diabetes.

A cross-sectional study of adult hypertensive patients in the United States found that high serum copper concentrations were significantly related to the risk of diabetes, indicating that copper could be an important risk factor for progression to diabetes. Furthermore, the association was more pronounced in certain populations, such as the better educated and obese, which implies that certain populations may be more susceptible to metabolic disorders (34).

The effect of copper on diabetes is not isolated, but may be influenced by a combination of other trace elements and metabolic factors. A study evaluating the role of gender and age factors in the relationship between micronutrients and diabetes by Wang et al. reported that serum copper levels were significantly associated with the incidence of diabetes in women, but a similar association was not observed in men. They hypothesized that this discrepancy may be related to differences in the metabolism of metallic elements in different sexes (35). Another study by Zhang et al. indicated that magnesium, copper, and FT4 levels are influential factors in the development of gestational diabetes mellitus (GDM), while maternal free triiodothyronine (FT3), free thyroxine (FT4), and FT3/FT4 ratios may be potential mediators of the association between metallic elements and the risk of GDM (36).

The pathogenesis of diabetes mellitus (DM) and its associated complications is significantly influenced by oxidative stress, which arises from excessive reactive oxygen species (ROS) production coupled with disruption of the cellular oxidative-antioxidant equilibrium (37). Under conditions of copper overload, Cu^+^ can participate in a Fenton-like reaction (Cu^+^ + H_2_O_2_ → Cu²^+^ + •OH + OH^-^) with hydrogen peroxide (H_2_O_2_), generating highly reactive hydroxyl radicals (•OH) and being oxidized to Cu²^+^. These hydroxyl radicals can induce protein oxidation, and DNA damage, ultimately leading to oxidative injury to cellular components (38). Furthermore, excess copper can inhibit the activity of antioxidant enzymes and induce excessive production of ROS, thereby promoting oxidative stress (39). This oxidative stress can directly damage macromolecules or indirectly lead to abnormal insulin secretion, decreased β-cell function and insulin resistance (40). From the perspective of inflammation and oxidative stress mechanisms, Pouresmaeil et al. conducted a comprehensive investigation into the correlation between copper, selenium levels, and T2DM. The research revealed significant associations between elevated copper and selenium concentrations and three critical metabolic parameters: impaired insulin sensitivity, enhanced oxidative stress responses, and upregulated inflammatory cytokine expression. This Studies have shown that inflammation and oxidative stress may influence changes in serum copper and selenium levels in patients with T2DM (41). Similarly, another regression analysis not only confirmed a positive association between serum copper and blood leukocyte levels but also revealed that inflammatory markers serve as a significant pathway through which copper influences blood glucose regulation (42). From another perspective, the results of a study by Menezes-Santos et al. on the effect of blood copper levels on glycemic control showed that copper-deficient T2DM patients have higher C-peptide concentrations and better β-cell function, and that lower copper levels may be more favorable for good glycemic control. Nevertheless, the study did not take into account the synergistic effect between copper and other micronutrients such as zinc (43).

However, a study conducted by Omidian et al. concluded the opposite. They recruited 40 patients with T2DM and metabolic syndrome (MetS) and 36 healthy controls and tested their blood for micronutrients. The T2DM groups were found to have significantly lower plasma copper, magnesium and zinc levels and lower erythrocyte copper levels than controls. This study concluded that changes in trace elements could be a significant factor in MetS and T2DM. In particular, measurements of trace element levels in erythrocytes appear to be of greater clinical value than serum or plasma samples, which have controversial results (44). Similarly, in a case-control study investigating the impact of copper, zinc, and selenium on T2DM, He et al. discovered that urban Chinese residents with higher serum levels of zinc and copper showed a reduced risk of developing the disease. Furthermore, they suggested a potential interactive relationship between serum levels of zinc-copper and selenium-copper in relation to T2DM risk. However, since the study was an exploratory analysis, the results only provide guidance for subsequent studies and need further validation (45).

The dual role of copper in the body may explain the conflicting results of these studies. While copper plays an essential role in supporting the function of antioxidant enzymes like SOD, elevated copper concentrations can conversely intensify oxidative stress through the increased production of ROS (40). Overall, the exact role of copper in glycemic control in diabetic patients is not well understood, mainly because of the complex mechanisms of interaction between the two. Moreover, the function of copper may be influenced by the interaction of other metabolic factors in the body. A study conducted in China revealed that elevated plasma copper levels were linked to a higher diabetes incidence among hypertensive adults with serum high-density lipoprotein cholesterol (HDL-C) concentrations of ≥1.2 mmol/L, indicating a potential involvement of HDL-C in the relationship between copper and diabetes (46).

Therefore, additional research is necessary to determine the optimal blood copper levels for diabetic individuals and to investigate its precise relationship with glycemic control, as well as the factors that may modulate this association.

### 3.3 Urinary copper and diabetes

Measurement of urinary metal concentrations, such as urinary copper concentrations, remains a widely used method for assessing metal burden and determining metal exposure in individuals, despite the different routes of excretion of different metals in the body (47, 48).

A Chinese cross-sectional study involving 2766 diabetic participants explored the potential associations between urinary levels of copper, zinc, arsenic (As), selenium (Se), strontium (Sr), and fasting plasma glucose (FPG). The results indicated that Zn levels were positively associated with FPG, whereas Cu and As levels showed negative correlations with FPG in women (49). Weiss et al. investigated the correlation between urinary metal concentrations and indicators of glycemic status in a Mexican-American population and found that elevated urinary levels of As, Se, Cu, molybdenum(Mo), nickel(Ni) and tin(Sn) were associated with a rapid increase in glycemic indicators and that higher urinary levels of copper were associated with lower pancreatic beta-cell function. This suggests that exposure to metallic elements such as copper and imbalances in essential metal homeostasis may accelerate the development of type 2 diabetes (50, 51). In contrast, Yang et al. carried out a cross-sectional study to examine the association between several urinary metal concentrations and the risk of DM in the Chinese Dong population. The results demonstrated that higher urinary Fe and Sr concentrations were linked to a reduced likelihood of DM, whereas urinary Cu concentration was not significantly associated with diabetes risk. The study also suggests that single metal exposure may not be sufficient to cause adverse health outcomes, while co-exposure to multiple metals may increase health risks, the exact mechanisms of which need to be further investigated in future studies (52).

There are still a number of confounding factors that need to be removed from studies of urinary copper and diabetes. Increased urinary copper concentrations may be due to increased urinary excretion rather than increased copper exposure. Furthermore, the balance of copper metabolism within the body, which may affect renal function, and kidney disease, which may affect urinary copper levels, should also be taken into account.

## 4 Copper and diabetic kidney disease

As a frequent complication of DM, DKD manifests clinically through proteinuria development/remission and accelerated glomerular filtration rate (GFR) deterioration (53). Due to the lack of effective prevention and treatment, DKD has become a leading cause of ESRD and death in diabetic patients (54). Recent years have witnessed a growing body of research exploring the association between copper and DKD.

### 4.1 The role of copper metabolism dysregulation in DKD

Copper homeostasis plays a crucial role in maintaining systemic physiological balance, and its dysregulation is closely associated with the pathogenesis and progression of DKD. In patients with DKD, disruptions in copper homeostasis primarily result from endocrine metabolic disorders, excessive dietary copper intake, and environmental heavy metal exposure (55). Current studies confirm that both copper deficiency and overload in diabetic individuals activate multiple signaling pathways (38).

Elevated copper levels can catalyze Fenton or Haber-Weiss reactions, promoting ROS generation and exacerbating oxidative stress, thereby leading to cellular damage or death (40). Notably, the recently proposed concept of “cuproptosis” reveals a novel regulated cell death mechanism. Unlike traditional oxidative stress-induced cell death, cuproptosis is primarily linked to lipoylation modification and oligomerization of tricarboxylic acid cycle-related proteins (38).

In the pathogenesis of DKD, metabolic disturbances and immune response-induced renal fibrosis are considered key pathological processes driving disease development. Cuproptosis-related genes have been demonstrated to modulate the immune microenvironment and metabolic pathways (56–58). Recent studies employing consensus clustering analysis based on cuproptosis-related genes (e.g., HRSP12 and DCXR) have further elucidated their association with DKD (59, 60).

**Figure 2:**
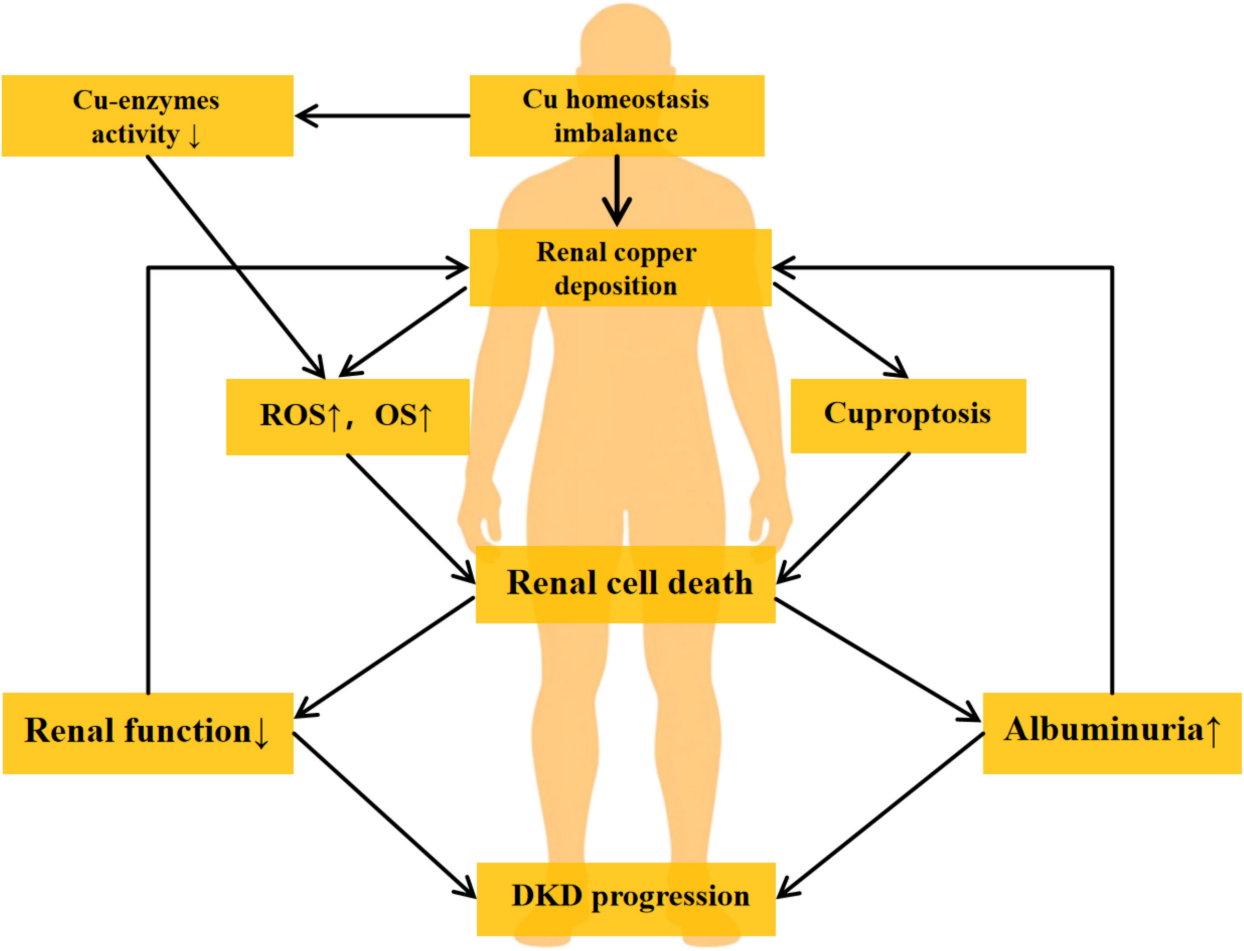
The vicious cycle of copper-induced renal injury in DKD. The diagram illustrates the proposed pathogenic loop linking copper dyshomeostasis and DKD progression. The cycle is initiated by hyperglycemia-induced systemic copper imbalance and renal copper deposition. Excess copper contributes to renal injury by inhibiting copper-enzyme activity, elevating ROS, and triggering cuproptosis, collectively leading to renal cell death and a decline in renal function. This dysfunction impairs copper excretion, while increased albuminuria may enhance tubular copper loading, thereby reinforcing copper accumulation and completing a self-sustaining vicious cycle that drives DKD progression. DKD, Diabetic Kidney Disease. ROS, Reactive Oxygen Species. OS, Oxidative Stress.

The relationship between copper and DKD is bidirectional. Clinically, serum copper levels are positively correlated with diabetes incidence and poor glycemic control. Hyperglycemia enhances ROS production and activates inflammatory pathways, indirectly contributing to renal cell damage (34, 61). Additionally, the diabetic state impairs the activity of copper-dependent antioxidant enzymes, exacerbating oxidative stress and inflammatory responses, thereby accelerating complication progression (8). On the other hand, copper overload leads to renal copper deposition, causing nephrotoxicity associated with interstitial injury and progressive renal dysfunction (62). Excessive copper also directly damages tubular cells (63, 64). Importantly, a vicious cycle may form between copper dysregulation and DKD: since the kidneys are a major excretory organ for copper (65), impaired renal function reduces copper excretion, exacerbating systemic copper accumulation and further aggravating kidney injury. Moreover, the increase in urinary albumin in DKD patients may promote the dissociation of albumin-Cu and ceruloplasmin-Cu complexes, elevating renal copper load and urinary copper excretion (66), thereby reinforcing a “copper toxicity-renal injury” positive feedback loop. The mechanism of copper in DKD is illustrated in **Figure 2**.

Given these mechanisms, copper metabolism dysregulation plays a pivotal role in DKD pathogenesis, providing a theoretical basis for novel therapeutic strategies. Studies have shown that copper chelators can mitigate renal injury progression in DKD by suppressing oxidative stress and collagen synthesis (67–69), demonstrating promising therapeutic potential.

### 4.2 Cuproptosis and DKD

Cuproptosis is a recently identified form of regulated cell death. It is characterized by the accumulation of Cu²^+^ within mitochondria, where it binds to acylated components of the tricarboxylic acid (TCA) cycle. This binding leads to the aggregation of acylated proteins and destabilization of iron-sulfur (Fe-S) cluster proteins, ultimately resulting in proteotoxic stress and cell death (70).

When excess copper ions influx into mitochondria, they directly bind to four lipoylated proteins involved in the TCA cycle: ① dihydrolipoamide S-acetyltransferase (DLAT), ② the α-ketoglutarate dehydrogenase complex (KGDC), ③ the glycine cleavage system protein H (GCSH), and ④ dihydrolipoamide branched-chain transacylase E2 (DBT). This binding induces their oligomerization, disrupting their function and impairing mitochondrial metabolism (71). Furthermore, the oligomerization of these proteins leads to decreased levels of Fe-S cluster proteins, which are crucial for the mitochondrial electron transport chain, thereby exacerbating mitochondrial dysfunction (72). Mitochondria generate ATP primarily through oxidative phosphorylation. Damage to mitochondrial function compromises this process, leading to insufficient ATP production, increased generation of reactive oxygen species (ROS), and ultimately, cellular damage and death (73).

Cu induces oxidative stress damage, protein acylation and oligomerization of acylated tricarboxylic acid (TCA) cycle proteins. These processes result in the downregulation of iron-sulfur cluster proteins and induce proteotoxic stress, ultimately disrupting cellular copper homeostasis and triggering cell death (74). Additionally, cuproptosis has been shown to significantly contribute to the progression of various renal diseases, including DKD (38). The introduction of this concept provides a new explanation for the mechanism of copper homeostasis in DKD.

### 4.3 Animal studies on copper and DKD

Gong et al. observed a significant elevation in renal copper levels in diabetic rats, leading to the hypothesis that impaired renal copper homeostasis may contribute critically to DKD progression (67). Pastacı Özsobacı et al. also reported a significant increase in renal copper levels in diabetic rats when compared to healthy controls. It was also accompanied by increased levels of oxidative stress, suggesting the presence of excess peroxides and hydroxyl radicals in diabetic kidneys, leading to impaired renal function. The study further pointed out that ACE inhibitors (ACEi) or AT1 receptor blockers could regulate trace element levels (e.g., zinc, magnesium, copper, and iron) and enhance renal antioxidant capacity, ultimately delaying DKD progression (75).

Recent studies have demonstrated that in diabetic mouse models, hyperglycemia upregulates the expression of lysyl oxidase (LOX) by activating the TGF-β signaling pathway. Upregulated LOX can induce partial epithelial-mesenchymal transition (EMT) in renal tubular epithelial cells, thereby promoting the development of renal fibrosis in diabetes. Further experiments have shown that inhibiting LOX activity effectively attenuates the EMT process in tubular epithelial cells, reduces the extent of renal fibrosis, and improves renal function parameters (76). It is noteworthy that LOX is a copper-dependent amine oxidase whose catalytic function is highly dependent on Cu²^+^ as a cofactor (77). Therefore, it is plausible that dysregulated copper metabolism may play a significant regulatory role in the progression of diabetic renal fibrosis by modulating LOX activity.

### 4.4 Clinical studies on copper and DKD

In clinical research, a cross-sectional study revealed that the urinary copper content in patients with DKD was significantly higher than that in non-diabetic kidney disease patients (78). Furthermore, Al-Bayati et al. recruited 55 patients with T2DM and divided them into microalbuminuria and normoalbuminuria groups, and 37 healthy patients as controls. The findings demonstrated that SOD levels were significantly reduced in the microalbuminuria group compared to healthy controls, a phenomenon strongly linked to elevated urinary copper excretion (79). In line with this, Talaei et al. observed a significant rise in urinary copper excretion among patients with microalbuminuria. They speculated that the increased urinary copper excretion may be related to the dissociation of copper carrier proteins (e.g. copper-albumin and ceruloplasmin-copper complexes) after passing through the impaired glomerular filtration barrier. However, the study does not exclude the possibility that urinary copper overload may contribute to accelerated progression of nephropathy in patients with advanced kidney disease. The limitation of this study was that serum copper and ceruloplasmin levels were not measured, which may have an impact on urinary copper excretion (80). In contrast, Ito et al. conducted a more comprehensive study comparing serum and urinary concentrations of copper, ceruloplasmin and albumin in 41 patients with type 2 diabetic kidney disease(T2DKD) and 10 healthy controls. The data showed that patients with macroalbuminuria exhibited markedly elevated urinary copper concentrations, while no significant differences were observed in serum copper levels across groups. It was concluded that the source of urinary copper in healthy individuals may be mainly the dissociation of albumin-copper complexes, whereas in patients with DKD urinary copper excretion may be due to the catabolism of copper-albumin and ceruloplasmin-copper complexes filtered through damaged glomeruli (66). According to the above findings, the increased urinary copper excretion in DKD patients may be related to urinary albumin, ceruloplasmin and impaired glomerular filtration barrier, but further damage to the kidneys by increased urinary copper excretion cannot be ruled out.

Clinical studies investigating the association between blood copper levels and DKD have still yielded inconsistent findings to date. 129 patients with T2DM and 128 healthy controls were recruited in the study by Ezzat et al. It was found that patients with DM and DKD had significantly higher serum copper levels, while serum zinc and magnesium levels were significantly lower compared to healthy controls. In addition to mineral elements, IL-17, TGF-β and miR-375 have also implicated its involvement in the pathogenesis of both T2DM and DKD (81). A study by Zaid et al. also revealed significantly increased serum copper levels in DKD patients compared to healthy individuals (82). Furthermore, to assess the effect of zinc on the role of copper *in vivo*, Takao et al. explored the relationship between blood copper/zinc ratio and inflammation and prevalence of DKD, and the results showed that an elevated copper/zinc ratio may exacerbate inflammation and synergistically correlate with a high incidence of DKD under inflammatory conditions (83). These findings underscore the significant contribution of copper to the development of DKD, while oxidative stress, inflammation and cytokines may also mediate the effects of copper metabolism on DKD.

But there are also studies that have come to the opposite conclusion. In their investigation of DKD patients, Sivaprasad et al. identified a strong positive association between plasma copper concentrations and eGFR, suggesting copper’s potential renoprotective effects. In addition, this study found that plasma micronutrient levels were not associated with dietary intake in patients and may be related to the influence of other factors such as physiological, hormonal as well as metabolic factors (84). A study by Makhlough et al. showed that serum copper, zinc and chromium (Cr) levels were significantly lower in diabetic patients with diabetic nephropathy compared to healthy controls. It was concluded that such changes may be attributed to factors such as urinary excretion, dietary micronutrient intake, absorption and utilization efficiency, and not solely determined by external factors such as water intake (85).

In addition, there are also studies that have found no significant association between the two. Prabodh et al. conducted a study comparing 40 DKD patients with 40 healthy controls, revealing that serum copper levels did not differ significantly between groups, whereas serum magnesium concentrations were substantially lower in the DKD cohort. It has been reported that magnesium deficiency may promote the development of vascular complications by increasing oxidative stress and decreasing insulin sensitivity in diabetic patients (86). In line with this, Temurer Afşar et al. investigated the association between T2DM microvascular complications and micronutrient levels and found that there were no significant differences in copper levels between groups, but decreased magnesium and chromium levels were correlated with T2DM microvascular complications (87).

Despite the progress made in animal and clinical studies in the field of copper and DKD, such as the finding of increased renal copper levels in diabetic rats and increased urinary copper excretion in DKD patients, the relationship between blood copper and DKD has not yet reached a unanimous conclusion and further studies are needed to clarify it. However, current researchers have revealed the relevance of other trace elements (e.g. iron, zinc, magnesium, selenium, etc.) with DM and DKD, which provides new ideas and directions to answer these inconsistent results. Furthermore, a study revealed that oxidized low-density lipoprotein (ox-LDL) may inhibit the copper transporter ATP7B, leading to copper overload and the induction of cuproptosis in renal tubular epithelial cells, thereby exacerbating lipid metabolism-related renal injury. This finding provides a novel perspective linking lipid metabolism to the association between copper dysregulation and kidney damage (88). Multi-center, large-sample clinical trials and mechanistic studies are necessary in the future to better elucidate the mechanisms of copper and other trace elements in DKD. The summary of clinical studies on the relationship between copper and DKD is shown in **Table 1**.

**Table 1:** Clinical studies on copper and diabetic kidney disease.

Study design, year	Subjects	Controls	Main results
Case-control study (Ito et al., 2001) (66)	41 patients with T2DKD: Group I: 15 diabetic patients with normoalbuminuria Group II: 14 diabetic patients with microalbuminuria Group III: 12 diabetic patients with macroalbuminuria	10 healthy subjects	Urinary copper concentrations were significantly higher in Group III compared with control group: 88.1 (11.0 – 174.2) vs. 20.1 (13.7 – 34.3) (p<0.001) There was no significant difference in serum Cu levels among the four group
Cross-sectional study (Talaei et al., 2011) (80)	42 T2DM patients with microalbuminuria	40 T2DM patients without microalbuminuria	Microalbuminuria group have significantly increased urinary copper excretion compared to the control group: 36.14 ± 10.8 vs.14.77 ± 2.3 (p=0.003)
Case-control study (Prabodh et al., 2011) (86)	40 patients with DKD	40 healthy subjects	There was no significant difference in serum Cu levels between the DKD group and the control group: 165.42 ± 5.71 vs. 166.6 ± 5.48 (p>0.05) There is no significant relationship between Cu levels and FBS, PPBS, HbA1c, and microalbumin concentrations
Cross-sectional study (Al-Bayati et al., 2015) (79)	55 T2DM patients: Group I: 31 patients in the microalbuminuria group Group II: 29 patients in the normoalbuminuria group	37 healthy subjects	The average urinary copper/creatinine ratio in the microalbuminuria group significantly increased compared to the control group: 53.3 ± 3.2 vs. 44.2 ± 5.3 (p<0.05) The SOD level in the microalbuminuria group was significantly reduced: 30.6U/ml vs. 45U/ml (p<0.05)
Case-control study (Makhlough et al., 2015) (85)	70 patients with T2DKD	70 healthy subjects	The average serum Cu level in T2DKD patients was significantly reduced (p<0.01) compared to the healthy control group
Cross-sectional study (Takao et al., 2021) (83)	651 patients with T2DM: 220 patients with DKD	No control group	The serum copper level in patients with DKD is significantly elevated compared to patients without DKD: 100.5 ± 15.5 vs. 97.0 ± 15.6 (P=0.007) The Cu/Zn ratio significantly increased in patients with DKD compared to those without DKD: 1.247 ± 0.265 vs. 1.155 ± 0.242 (p<0.0001)
Cross-sectional study (Ezzat et al., 2023) (81)	129 patients with T2DM: 39 patients with microalbuminuria 41 patients with macroalbuminuria 49 patients without kidney disease	128 healthy subjects	The serum Cu level in the T2DM group significantly increased compared to the control group: 223.4 ± 42.2 vs. 186.8 ± 57.3 (p< 0.05) The serum Cu levels in the macroalbuminuria group were significantly elevated compared to the group without DKD: 249.5 ± 46.3 vs. 218.5 ± 34.6 (p< 0.05)
Cross-sectional study (Temurer Afşar et al., 2023) (87)	118 patients with T2DM: Group I: 40 patients without microvascular complications Group II: 38 patients with only retinal lesions Group III: 40 patients with retinal lesions and kidney disease	No control group	There was no significant difference in Cu levels between the groups: Control: 16.3(4.49-31.4) vs. Group I: 15.5(5.8-33.1) vs. Groupe II: 15.8(7.9 -31.3) vs. Group III: 16.5(6.5-1027.0) (p=0.301)
Case-control study (Zaid et al., 2024) (82)	60 patients with DKD	60 healthy subjects	The average serum Cu level in the DKD group significantly increased compared to the control group: 144.20 ± 10.60 vs. 70 ± 13.75 (p<0.05)
Case-control study (Sivaprasad et al., 2024) (84)	Group I: 74 patients with DKD Group II: 66 diabetic patients without chronic kidney disease	54 healthy subjects	The plasma Cu levels in the DKD group were significantly reduced compared to the control group: 154 (113–186) vs. 176 (149–204) (p=0.005)
Cross-sectional study (Gao et al., 2024) (78)	258 diabetic patients with mid DKD 140 diabetic patients with end DKD	432 diabetic patients without DKD	The concentration of urinary Cu and Mn was higher in DKD than in NDKD, and higher in end DKD: NDKD: 209.0(110.6-373.3) vs. Mid DKD: 223.6(132.2-405.2) vs. End DKD: 305.8(183.9 -435.2) (p<0.001)

Cu, copper; T2DM, type 2 diabetes mellitus; DKD, diabetic kidney disease; T2DKD, type 2 diabetic kidney disease; NDKD, non-DKD; FBS, fasting blood sugar; PPBS, postprandial blood sugar; HbA1c, glycated hemoglobin; SOD, Superoxide dismutase.

### 4.5 Copper chelator and DKD

The therapeutic application of copper chelators was previously established in Wilson’s disease treatment and relevant studies have shown that they are also able to prevent or reverse diabetes and its complications, such as DKD, by inhibiting diabetes-associated copper overload and oxidative stress (89).

Triethylenetetramine (TETA) is a potent and highly selective copper chelator (90), and has shown beneficial effects in the treatment of DKD. A study conducted by Gong et al. found that TETA was able to significantly reduce the copper content in the kidneys of diabetic rats, decrease the elevated urinary albumin level and inhibit the development of renal fibrosis. Notably, TETA treatment did not significantly alter blood glucose levels in diabetic rats, indicating that its renoprotective effect may be independent of the glucose-lowering mechanism, which suggests that TETA may be clinically valuable as an adjunctive treatment for DKD management (67). Lu et al. further investigated the efficacy of TETA in rats suffering from diabetic cardiac and renal dysfunction, and the results showed that it may enhance the body’s antioxidant defense mechanism by regulating the balance of copper metabolism to limit diabetes-induced cardiac and renal damage. It also enhances antioxidant defenses by increasing superoxide dismutase activity. Conversely, none of the other two less selective copper chelators (D-penicillamine and desferrioxone) or zinc acetate (which reduces copper absorption via competitive inhibition) had any effect on diabetes complications. This result indicates that TETA’s cardiorenal protective effects against diabetes-induced damage may be mediated through its high copper(II) binding specificity (68).

Moya-Olano et al. also compared the efficacy of the copper chelator trientine (triethylenetetramine (TETA) dihydrochloride) with renin-angiotensin system (RAS) blockers on the progression of glomerular pathology in diabetic rats. The results of the study showed that trientine significantly reduced proteinuria and albuminuria, inhibited the increase in creatinine clearance and kidney weight, and improved diabetes-associated glomerulopathological features in diabetic rats. Compared with RAS blockers, trientine also slowed glomerular cluster-capsule adhesion and reduced the incidence of tabularization. The protective mechanism of trientine has been hypothesized to be that it lessens glomerular cluster-capsule adhesion, decreases tabularization and maintains glomerular structural integrity by facilitating urinary copper excretion and reducing excess extracellular copper loosely bound to extracellular matrix components. The mechanism may represent a unique advantage of trientine over existing therapies in the treatment of DKD (69).

## 5 Discussion and conclusions

The review of the relevant literature shows that the relationship between copper and DM has gradually gained attention and some progress has been made in different countries. However, there are significant inconsistencies and even contradictions in the results of existing studies.

Research on copper-diabetes relationships has primarily investigated the link between diabetes risk and dietary copper consumption, but results have been inconsistent due to possible recall bias in dietary surveys and individual differences in copper intake, absorption and metabolism. Previous studies have been limited to dietary surveys. Future research could attempt to provide intervention guidance for patients’ diets and measure levels of blood copper, urine copper, etc., in order to assess the impact of dietary intervention on circulating copper levels. This would strengthen the causality and reliability of dietary research, potentially contributing to the progress of related studies.

For these reasons, more studies have been done into the correlation between blood copper levels and diabetes. In all the studies reported in this review, most of them showed that blood copper levels were significantly positively correlated with diabetes. However, a few studies found that it was negatively correlated and unrelated, and this contradiction may be due to these reasons: 1. There may be a threshold effect or U-shaped curve relationship between blood copper and diabetes. A cross-sectional study identified a nonlinear inverted U-shaped association between fasting plasma glucose (FPG) levels and urinary copper excretion (91). Another study also suggested a U-shaped dose-response relationship between dietary copper intake and the risk of diabetic retinopathy (92). From a physiological mechanistic perspective, copper acts as an essential cofactor for antioxidant enzymes such as superoxide dismutase (SOD), exerting antioxidant effects within an appropriate concentration range. However, excess copper can promote the generation of reactive oxygen species (ROS), thereby exacerbating oxidative stress. Given that both copper deficiency and overload exert adverse effects on human health, these findings support the biological plausibility of a U-shaped relationship between copper status and diabetes (93). Nevertheless, further research is required to elucidate its precise characteristics. 2. The results of the study may be influenced by various confounding factors, and other metal elements (zinc, iron), thyroid hormones (FT3/FT4), blood lipids (HDL), and inflammatory factors (IL-17) may act as effect modifying factors, which need to be further explored and rigorously corrected in the study. 3. Most of these studies have detected the level of copper in serum or plasma. In view of the controversial results, it may be a better choice to try to measure copper in red blood cells, and more information about copper metabolism in diabetes patients can be obtained (94).

In the field of DKD, researchers have found significantly increased renal copper levels in diabetic rats in animal studies and increased urinary copper excretion in patients with DKD in clinical studies. This may be related to the dissociation of copper-albumin or copper-ceruloplasmin complexes across the impaired glomerular filtration barrier, and the potential role of renal copper overload in the progression of nephropathy cannot be excluded. However, the number of studies on blood copper and DKD is insufficient, and the results are still controversial. In addition to the effects of other trace elements, factors such as patient age, renal function, and urinary albumin levels may also have a certain impact on the study of copper and DKD. More prospective studies are still needed to explore their relationship. Nevertheless, copper chelating agents, ACEI, ARB and other drugs have been proved to improve the progress of DKD by reducing the deposition of copper in the kidney, decreasing albuminuria, improving the pathological characteristics of DKD, enhancing antioxidant defense and other benefits, which is worthy of further clinical trials.

In general, the inconsistency of current research results on copper, diabetes and DKD shows that the role of copper in the development of diabetes may be more complex than expected, and more research is needed to reveal its potential mechanism and provide scientific basis for clinical intervention.
